# Does Intraindividual Variability of Personality States Improve Perspective Taking? An Ecological Approach Integrating Personality and Social Cognition

**DOI:** 10.3390/jintelligence6040050

**Published:** 2018-11-27

**Authors:** Richard Wundrack, Julia Prager, Eva Asselmann, Garret O’Connell, Jule Specht

**Affiliations:** Department of Psychology, Humboldt-Universität zu Berlin, 10099 Berlin, Germany; julia.prager.1@hu-berlin.de (J.P.); asselmae@hu-berlin.de (E.A.); garret.oconnell@hu-berlin.de (G.O.); jule.specht@hu-berlin.de (J.S.)

**Keywords:** big five personality traits, personality states, intraindividual variability, whole trait theory, perspective taking, theory of mind, egocentric bias, simulation, anchoring, adjustment

## Abstract

Research integrating cognitive abilities and personality has focused on the role of personality traits. We propose a theory on the role of intraindividual variability of personality states (hereafter state variability) on perspective taking, in particular, the ability to infer other peoples’ mental states. First, we review the relevant research on personality psychology and social cognition. Second, we propose two complementary routes by which state variability relates to anchoring and adjustment in perspective taking. The first route, termed ego-dispersion, suggests that an increased state variability decreases egocentric bias, which reduces anchoring. The second route, termed perspective-pooling, suggests that an increased state variability facilitates efficient adjustment. We also discuss how our theory can be investigated empirically. The theory is rooted in an ecological interpretation of personality and social cognition, and flags new ways for integrating these fields of research.

## 1. Introduction

Are you ever struck by how diverse you can be in everyday life? Consider solely your extraversion within the scope of your daily work as a researcher; you may be talkative in your lectures, quiet during team meetings, outgoing with participants, yet coy at scientific conferences. Experiencing such diverse manifestations of your personality may have a positive side effect when putting yourself in someone else’s shoes. On the one hand, experiencing the fleeting nature of your own perspective might help you to distance yourself from your own current perspective when considering another one. On the other hand, experiencing the diversity of your own personality manifestations might help you to skillfully construct another person’s perspective.

Here, we propose a theory suggesting that such a diversity in personality manifestations, captured by the within-person variability of personality states (hereafter state variability), is positively associated with perspective taking, the ability to infer other people’s mental states. The theory posits two routes for this effect. One is an effect of state variability on perspective taking through changes in egocentric bias—the tendency to overestimate the relevance of your own current perspective. This route we have termed ego-dispersion. According to ego-dispersion, individuals higher in state variability have a lower egocentric bias, which allows them to consider other people’s perspectives while being less restrained by their own perspective. The other route is an effect on the skill to construct the mental states of others. This route, we term perspective-pooling. According to perspective-pooling, individuals higher in state variability have a wider repertoire of self-experienced perspectives that they can apply when reconstructing someone else’s perspective.

Ackerman argues in this issue that there are “relatively few hypotheses that address the *how* question” (italics in original, [1]) concerning the relation between personality and intellectual abilities. With our theory, we propose an answer to such a question, “how does state variability influence perspective taking abilities beyond the influence of personality traits?”. Before presenting the theory in more detail, we outline the research forming the basis for our theory and guiding its future, empirical investigation.

## 2. Literature Review

### 2.1. Personality Psychology

#### 2.1.1. Personality Traits and States

Personality traits describe the individual differences in people’s general patterns of thoughts, feelings, and behaviors that stay relatively stable over time. A large proportion of these differences can be described by the Big Five personality traits, namely: openness to experience, conscientiousness, extraversion, agreeableness, and emotional stability [2]. The Big Five have been shown to reliably predict a variety of life outcomes, including academic performance and subjective well-being, among others [3,4,5,6,7]. While the Big Five have shown to be reliable over time, it needs to be noted that these traits are specifically conceptualized to capture temporally stable between-person differences in personality, and that they are insensitive to the moment to moment within-person variability of thoughts, feelings, and behaviors [8,9]. The everyday life fluctuations in personality manifestations are described by personality states.

**Figure 1:**
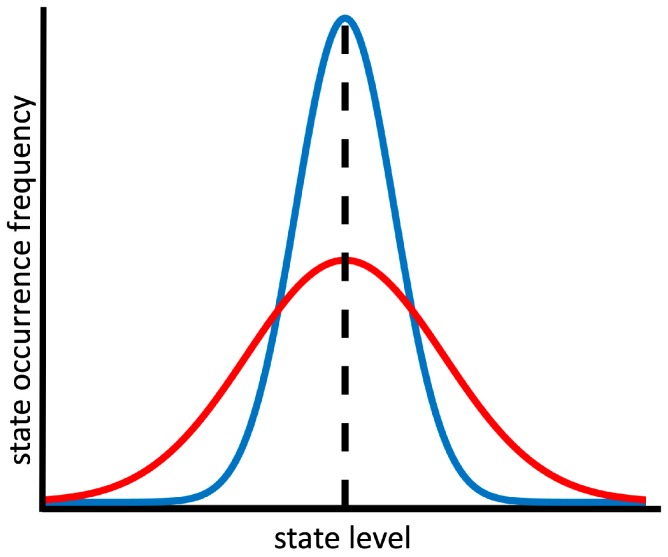
Personality state density distributions. The red and blue line describe the state density distributions corresponding to a personality trait of two individuals (cf. state extraversion of Red and Blue in the example given in the text). The dashed line specifies the mean shared by both distributions.

Whole trait theory is a prominent model that integrates the trait and state approach to personality [10]. Here, personality states are simply defined “as having the same affective, behavioral, and cognitive content as a corresponding trait but as applying for a shorter duration” (p. 84, [10]). Fleeson and colleagues’ research [9,10,11,12] suggests that everyone will eventually express the entire range of possible personality state levels, but that there are individual differences in the frequency with which the different state levels occur in everyday life. This is expressed by the density distribution of personality, which you get if you plot the state occurrence frequency as a function of the state level (cf. Figure 1) (note that in real-life, density distributions are not necessarily normally distributed). It can also be read as an individual’s trait-specific disposition to be in one personality state as compared to another. The mean point of such a density distribution is taken to describe the state-corresponding trait level, while the variance around the mean (e.g., standard deviation) describes the state variability.

Imagine that the two curves in Figure 1 describe the state density distributions of extraversion for two people, Red and Blue. Their density distributions have the same mean (dashed line), therefore Red and Blue have the same level of trait extraversion. However, Blue’s state level extraversion is more narrowly distributed than Red’s (i.e., Blue’s state variability is lower). That means that Blue experiences fewer instances of more extreme manifestations of extraversion—in this case, in both directions. We argue that this would put Red at an advantage over Blue when considering other people’s perspectives, because Red is more experienced with a wider range of perspectives, as they accompany the more diverse manifestations of Red’s extraversion.

#### 2.1.2. State Variability and Self-Reports on Personality States

State variability is captured by the variance of an individual’s trait-correspondent density distribution. It has been argued that state variability may be a global trait in itself [13], that is, if a person is variable in one trait, they are likely to be variable in other traits as well. However, there is an ongoing debate regarding the confounds of intraindividual variability measures that question that state variability is a global trait. As this issue is currently unresolved, here, we consider state variability as a stable feature of the separate traits.

As illustrated by the example of Red and Blue (cf. Figure 1), the personality trait level and state variability should be two independent aspects of a density distribution. However, practical concerns were raised with respect to the repeated self-report measures of personality states on which most research on intraindividual variability is based [13,14,15,16]. For example, if a person scores extremely low or high on a given personality trait, floor- or ceiling-effects must occur because of the limits of the utilized scale, restricting the ability to measure changes in state variability at these extreme endpoints. Therefore, a mean-corrected variance parameter should be used as an indicator of state variability.

Fortunately, Mestdagh and colleagues recently introduced a widely applicable correction, the relative variability index, which is defined as “the ratio of the variability divided by the maximum possible variability given the mean” (p. 5, [16]).^1^ Unfortunately, correcting for the confound with the mean led Baird, Le, and Lucas [13] to conclude that the previously established relationships of state variability with psychological maladjustment and lower subjective well-being [17,18,19,20,21] vanished. Thus, they argue that state variability may be of no predictive value beyond trait levels. Mestdagh and colleagues raised similar concerns regarding the variability of emotional instability, questioning its utility as a diagnostic feature of borderline personality disorder [16].

In a follow-up paper, Baird, Lucas, and Donnellan [22]^2^ highlight yet another confound of state variability based on repeated self-report measures, as follows: the measured variance might largely be a reflection of an individual’s response style, that is, their tendency towards mild or extreme responses on questionnaire scales. With supporting evidence beyond the Big Five, they argue that the concept of intraindividual variability in general, as it is currently assessed and operationalized, is a confounded construct whose usefulness has yet to be shown.

In response, Deng and colleagues [23] developed a correction for extreme response styles by extending Bock’s [24] nominal response model, and Zettler and colleagues [25] recently proposed yet another approach that corrects not only for extreme but also indifferent and directional response styles. After correction for both mean and response style, Deng and colleagues were able to show the improved predictive power of intraindividual affect variability concerning smoking cessation [23]. Thus, their work suggests that intraindividual variability can be meaningfully related to other psychologically interesting constructs if the necessary care is taken.

#### 2.1.3. Complementing Self-Reports with Behavioral Measures on Personality States

Taken together, the above concerns give reason for researchers interested in state variability to complement self-reports of personality traits in a multi-method approach with measures of well-defined behaviors, which are not prone to the mentioned confounds^3^. Behavioral measures provide objective information that is not distorted by an individual’s explicit self-concept. For example, given an ecological momentary assessment of personality states [26,27,28,29], audio snippets from people’s daily lives—recorded with the electronically activated recorder (EAR) application [30,31], transcribed, and analyzed with the linguistic inquiry and word count (LIWC) software [32]—could be used to match an individual’s recorded talkativeness proximate in time to their self-reports on extraversion, and their positive word use proximate in time to their self-reports on agreeableness. This would allow for comparing the self-experienced and objective levels of state extraversion and agreeableness. Of course, similar matches have to be found for each personality trait that one is interested in, for example, all of the Big Five personality traits.

Importantly, it is not enough to gather only behavioral data. Behavioral measures capture a diversity of experience that does not equal the diversity of perspectives with respect to these experiences. Consider state variability—the continuous change in temporary thoughts, feelings, and behaviors—a product of the diversity of experience and the diversity of perspectives as they are relevant to personality. The diversity of experience as it is understood here is the diversity of objective experience—who does what, when, where, how, and with whom. This can be assessed with naturalistic behavioral measures and life-logging [33]. Diversity of perspective, on the other hand, describes the diversity of subjective experience of how someone actually thinks and feels, while being engaged in those objective experiences. Currently, the most ecological assessment method in that regard is from repeated self-reports given by the participants as they go about their lives [26,27,28,29].

While it is certainly the case that the diversity of experience is positively associated with the diversity of perspectives, it can only be a coarse approximation. The behavioral measures that capture the objective experience cannot capture the accompanying variability in thoughts and feelings as captured by repeated self-report measures of personality states. A good example comes from an approach to state variability that makes a further distinction between within- and cross-context variability [15]. On the one hand, state variability can occur across contexts, for example, Red is extraverted with friends but introverted with colleagues. On the other hand, variability can occur within contexts, for example, Blue is sometimes extraverted and sometimes introverted when surrounded by friends. Theoretically, both kinds of variability can add to the state variability we are interested in; thus, at this point, we do not make any strong claims about which relation to context is more relevant to our theory. However, it highlights how an exclusive reliance on a behavioral measure might fall short in capturing the diversity of perspectives with respect to within-context variability. For example, imagine estimating Red and Blue’s extraversion based on the number of their interaction partners. What you might miss, however, is that Blue might feel increasingly uneasy as the group size increases, while Red enjoys interacting with some groups but not with others.

Thus, to counterbalance the respective shortcomings of behavioral measures and self-reports, and to capture trait-specific state variability, we suggest a multi-method approach to state variability, combining repeated self-report and behavioral measures into a single state variability estimate, for example, by confirmatory factor analysis. Furthermore, with respect to the last example, we also recommend expanding the ambulatory assessment of personality states by measures like the ultra-brief measure for the situational eight DIAMONDS domains [34,35], in order to differentiate between contexts or situations.

### 2.2. Social Cognition

#### 2.2.1. Empathy and Perspective Taking

The success of social interactions depends on our mutual understanding. However, this is not a unitary ability. Prominently, it involves the ability to share others’ feelings (i.e., empathy) and the ability to infer others’ mental states (i.e., perspective taking (also theory of mind or mentalizing), among others). Historically, the distinction has not always been straightforward [36]. We follow the modern distinction of Preckel, Kanske, and Singer, who define empathy as a socio-affective “process of sharing feelings, that is, resonating with someone else’s feelings, regardless of valence (positive/negative), but with the explicit knowledge that the other person is the origin of this emotion” [37]. In contrast, perspective taking is a socio-cognitive process of inference and reasoning about someone else’s beliefs, thoughts, or emotions, that results in propositional knowledge about their mental state [37]. This conceptual distinction mirrors brain imaging research, suggesting distinct neural networks underlying both processes [38,39,40].

Understanding other people’s mental state in real-life is a complex task and may require considerations of their circumstances, beliefs, knowledge, feelings, intentions, and their personality [41]. In doing so, motor empathy [42], the automatic mimicking of and synchronization with another person’s motor output—their posture, movements, facial expression, and vocalizations—aids both the sharing of their feelings and the understanding of their mental states [43,44,45]. However, most of the standard perspective taking tasks [46,47,48,49,50] do not require such holistic reasoning efforts but focus solely on what the other can know and what they are going to do. Moreover, they are not designed to allow for motor empathy to improve performance, because only pictographic and static scenes [46,47,48,49,50] or short clips are presented to the participants [51].

Take, for example, the classic perspective taking task, the false-belief task developed for autistic children [52]. In this task, two dolls, Anne and Sally, are presented to a child. Sally puts a marble in a basket and leaves the scene. Anne takes the marble out of the basket and puts it into a box without Sally’s knowledge. When Sally comes back looking for her marble, the child is asked where Sally is going to look for her marble, in the basket or in the box. In this task, the possible feelings and personalities of the dolls are irrelevant, nor can the child make use of motor empathy to understand Sally or Anne’s behavior.

**Figure 2:**
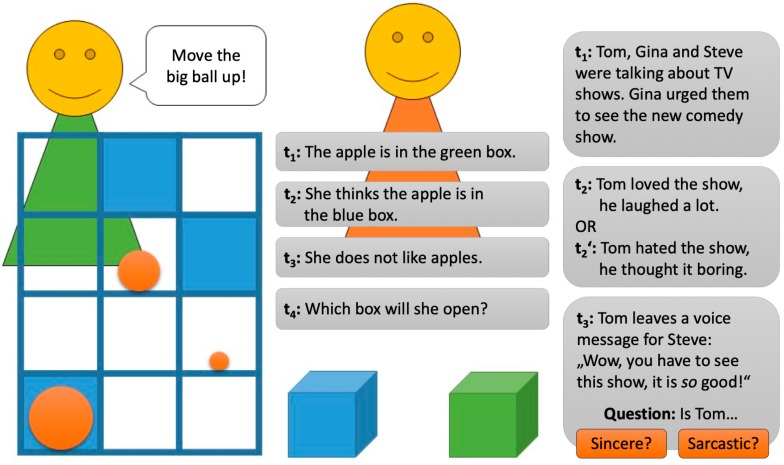
Standard perspective taking tasks. From left to right: the director’s task, belief–desire continuity test, and adapted anchor and adjustment paradigm.

Of course, more sophisticated perspective taking tasks have subsequently been developed for adults [37,38,39,40,41,42], but they usually share similar limitations. Consider Figure 2 from left to right. During the director’s task [46], the participant has to account for a director’s limited knowledge because of the director’s limited field of vision while following the instructions to move certain objects. Similar to the false-belief task, the belief–desire continuity test [26] requires participants to make informed guesses regarding where someone else will look for a desired object. Another paradigm [47] requires participants to determine the intention communicated in a voice message.

Another, more naturalistic assessment of perspective taking that is not depicted here is movie for assessment of social cognition (MASC) [51], in which social inferences have to be made about characters engaged in a discussion, which is shown in a short movie clip. Assessments based on video clips and standardized interactions in virtual reality—although the latter has so far mostly been used to train and not to test social cognition in autism [53,54]—are currently the most ecologically valid approaches to investigating perspective taking more holistically. To investigate our theory, we suggest implementing a perspective taking task that requires participants to make social inferences of personality trait-relevant content (i.e., thoughts, feelings, and behaviors that are connected to different personality traits). Further key features of the required task will be specified in the following section.

#### 2.2.2. Perspective Taking as Simulation

According to simulation theory, for most real-life cases of perspective taking, we can apply self-knowledge to make the right social inferences [55]. Simulation theories suggest that we can imitate, copy, or reproduce other’s perspectives based on our own mental experiences [56]. Goldman [57], for example, holds that before attributing a perspective to others, we generate and introspect a model perspective. Simulation theory further suggest that perspective taking involves the two processes of anchoring and adjustment, which are crucial to our theory.

Anchoring is often only considered in terms of establishing an initial anchoring perspective from which a simulation starts off—the anchor is often a person’s own current perspective. However, we consider it much more useful to think of anchoring as a person’s readiness to deviate from their initial perspective when simulating someone else’s perspective. Adjustments are made serially to the initial perspective until an acceptable approximation of the other person’s perspective is reached. How fast and accurate we can make these adjustments depends on, among others, on how much we can rely on our self-knowledge (i.e., memories and familiar thoughts, feelings, and beliefs we associate with the circumstances we deem the other person to be in). However, at which point the adjustment process is terminated—which eventually determines how accurate we will be—depends on how much our anchor holds us back from deviating from our own perspective. Thus, anchoring and adjustment are interlocked; the larger the initial self–other discrepancy in perspectives, the more one has to adjust. But how accurately one adjusts depends first on the information a person can draw on, and second, at which stage of the simulation they stop to adjust.

For our purposes, the perspective taking task has to be able to differentiate between the anchoring and adjustment effects. We suggest a speed–accuracy task similar to Tamir and Mitchell’s design [55], where subjects report their own perspectives before they infer those of others, to allow for parameterizing the initial and final perspective and the self–other discrepancy in the perspectives. The anchoring effect can be operationalized as the relative difference in the initial and final perspectives (i.e., the actualized readiness to deviate (adjust away) from one’s own perspective). To the extent that an individual is less anchored in their own perspective, they should in comparison account for more self–other discrepancy. To the extent that participants simulate more efficiently, they should be able to adjust faster for a given self–other discrepancy in perspectives. Finally, only if both the anchoring and adjustment are improved, perspective taking should be more accurate (i.e., the final self–other discrepancy in perspectives should be minimal).

#### 2.2.3. Egocentric Bias

The ability to differentiate between one’s own mental states and those of another person is crucial for perspective taking. A failure to do so can stem from egocentric bias; the tendency “to project one’s own emotional or mental states on someone else” (p. 3, [37]). For example, you might mistakenly assume that because you cherish a tidy workspace, your colleagues do too.

Another way to think of egocentric bias is the (overly) self-referential structuring of information [58]. From this perspective, overcoming egocentric bias means to account for this dynamic by actively distancing yourself from your own perspective, and by disregarding your immediate feelings, knowledge, beliefs, and intentions (note, that merely overcoming egocentric bias does not necessarily mean that you are better at perspective taking [59]; importantly, you have to do so in favor of what you know about the other person). In fact, one may think of egocentric bias as a generic overestimation of the immediate over the distant—temporally, spatially, and socially. For example, it has recently been argued that overcoming the egocentric bias involves the same processes when considering others and considering a future or past version of oneself [60,61,62,63]. Notably, O’Connell and colleagues [60,61] suggest that overcoming egocentric bias when thinking about one’s future self and about the perspective of others is regulated by the same neural network. This supports the idea that egocentric bias is a general feature of our cognition, to overstate the immediate over the distant. This begs the question of whether people who score higher in state variability are better at perspective taking, because they experience more diverse thoughts, feelings, and behaviors more regularly themselves (i.e., these thoughts, feelings, and behaviors are more immediate or less distant to them).

### 2.3. Linking Personality and Cognition

#### 2.3.1. Personality Traits and Perspective Taking

Aside from a recent study suggesting social cognitive advantages for individuals with flexible personalities styles [64], previous research has often focused on relating personality and empathy in the context of medical practice and patient satisfaction [65,66,67,68,69]. For example, Song and Shi [69] analyzed the answers of 530 Chinese medical students on a Big Five Inventory and the Interpersonal Reactivity Index, an empathy questionnaire with four dimensions, one of which is perspective taking [70]. They found that perspective taking was moderately associated with agreeableness, while it was modestly associated with neuroticism, openness, and conscientiousness, accounting for 19.4% of the variance.

In fact, all of the Big Five personality traits have at some point been shown to have some meaningful relationship to perspective taking [65,66,67,68,69], and there are intuitions for all of them. Open people might encounter more diverse situations, which makes them knowledgeable about more perspectives and situations (cf. Section 2.3.2). Conscientious people might be more intent upon taking all of the necessary steps. Extraverted people might be engaged in more social interactions, exposing them more to the viewpoints of others. Agreeable people may be more motivated to understand the other person, because they strive for a harmonious relationship. Finally, emotionally stable people might be less anxious and thus less egocentrically biased when taking someone else’s perspective [50]. Given (a) the widespread association of personality traits and perspective taking, and (b) the possible confounds of trait levels and state variability discussed above [13,16], investigators are well-advised to be particularly sensitive to the impact of personality trait levels on perspective taking, independent of state variability. Furthermore, the widespread associations suggest that it is worthwhile investigating the state variability of all of the traits with respect to perspective taking and thus our theory. Moreover, possible relations to empathy could be considered as well.

#### 2.3.2. State Variability, Openness, and Intelligence

Research on the relationship between openness to experience and intelligence (e.g., the openness-fluid-crystallized-intelligence (OFCI) model [71]), has inspired this paper’s central claim, that personality can influence cognitive abilities. As in the OFCI model, we understand fluid intelligence as “to use deliberate and controlled mental operations to solve novel problems that cannot be performed automatically” (p. 5, [72]). Ziegler and colleagues’ [71] investigation explored the mutual influence of openness and fluid intelligence. On the one hand, they investigated the environmental enrichment hypothesis, wherein people who score high in openness may be exposed to more novel, intellectually stimulating situations, which positively influence their fluid intelligence [73]. On the other hand, they investigated the environmental success hypothesis, wherein having a higher fluid intelligence enables people mastering novel situations, which might make it more likely for them to be more open and seek more novel, mentally challenging situations [74,75].

In brief, the author’s findings support the former but not the latter hypothesis [71], that is, they find support that a personality trait can affect cognitive abilities by enriching the stimulation of the mind via the environment. This is in line with our notion of the diversity of experiences. The current proposal expands this notion, by arguing that the personality states themselves are stimulating multipliers of experiences (i.e., subjective experiences). Therefore, we suggest that the assessment of state variability has to comprise the diversity of perspectives. Open people may explore more novel situations (i.e., increased diversity of experience), but individuals high in state variability will experience a more diverse set of perspectives across situations (i.e., diversity of perspectives).

The OFCI model is relevant in at least two more ways. First, assuming that goals and motivations (seeking more intellectually stimulating situations) can be a production mechanism for personality traits (openness to experience) [76], one might also want to consider that contemplating the perspective of others might affect state variability. This is not predicted by our theory but could be tested by evaluating whether training in perspective taking increases state variability. Second, their two hypotheses may apply similarly to our case, making openness and fluid intelligence competing influencers of perspective taking independent of state variability. In line with the environmental enrichment hypothesis, open people may be better at taking other people’s perspective, because of their extensive experience with different situations. In line with the environmental success hypothesis, intelligent people may be more successful in making social inferences, motivating them to make even more social inferences, which improves their overall perspective taking abilities.

Moreover, there is evidence that intelligence is positively associated with perspective taking [77,78]. Fluid intelligence may be particularly relevant to adjustment, which, in contrast to anchoring, is a more readily controlled mental operation. Thus, not only do we recommend to control for the influence of personality trait levels, but for that of fluid intelligence on perspective taking as well, (e.g., by including an intelligence test like the I-S-T 2000 R [79]). In contrast, crystalline intelligence, understood as acculturated knowledge [72], appears to be less relevant to perspective taking—at least when cultural differences do not play a major role for successfully taking someone else’s perspective.

### 2.4. Intermediate Conclusion

In reviewing the literature on personality and social cognitive psychology, we set the stage to ask and answer the question of whether and how state variability may influence perspective taking. With respect to personality psychology, we argued that whole trait theory offers an intriguing approach, in which state variability is a feature of traits that await more thorough investigation. However, we also highlighted the methodological issues of operationalizing state variability. With respect to social cognition, we differentiated the roles that egocentric bias, anchoring, and adjustment play for the efficient perspective taking. Research joining both fields is rather limited and awaits more extensive investigations (e.g., in the light of our theory presented below). Furthermore, research on openness and fluid intelligence gives reason to believe that state variability can affect perspective taking by diversifying our perspectives. Thus, based on the reviewed constructs and ideas, we propose two routes by which state variability may influence perspective taking.

## 3. Two Routes: Ego-Dispersion and Perspective-Pooling

Up to this point, we have assumed a certain interchangeability of the contents of personality states and perspectives by operationalizing the diversity of perspectives as the state variability of repeated self-reports. The argument in favor of such an approach is that the content of self-reports in personality state assessments overlap with those during naturalistic perspective taking. In both cases, we are interested in a person’s thoughts, feelings, and (intended) behaviors. Thus, we argue that self-reports on personality states capture essential parts of a person’s perspective, the subjective experience of their inner life, its outer expression, and their relation to the world. If self-reports on personality states approximate people’s perspectives, their state variability may also hold information relating to the diversity of their perspectives. Notably, there is no reason to believe that this is limited to social traits. One’s perspectives will be diversified by the extent to which one engages with the world at different state levels of extraversion, as well as different state levels of conscientiousness.

In addition, we argue that a person’s own perspective can inform their reasoning regarding another’s perspective. Therefore, we propose that increased state variability improves perspective taking. Imagine, doll Sally from the classic false-belief task was to report on her personality state just before she goes back into the room with Anne and her marble. Sally may report that she is distrustful, which explains why she looks for the marble she had just put away. There are two ways in which having high personality state variability might help us to accurately infer Sally’s suspiciousness from her behavior. As we experience a wider range of trust-distrust beliefs and feelings in our own lives, two things occur. Firstly, our trust state variability will be rather distributed, indicating that our disposition to be trustful is biased away from a specific interpretation of the situation. Thus, we are freer to choose an interpretation based on other factors such as contextual cues, for example the hasty return of Sally to the room. In contrast, if you are by nature an extremely trusting person, you may miss this cue. Moreover, if you were extremely distrusting, your advantage in this specific set-up would be coincidental. We term the diminished egocentric bias that weakens anchoring because of the experience of a wider range of possible dimensions of a given state ego-dispersion.

Secondly, familiarity with the situation might facilitate fast and accurate perspective taking via another route. If we happened to have been in a comparable situation (i.e., storing something of value while someone else is watching us suspiciously), it is more likely that we will correctly predict Sally’s mistrust. Even if we were currently very trusting, having previously experienced a similar situation would facilitate the adjustment of our perspective. In contrast, if we were trusting by nature and had never been in a situation wherein we felt spied on, we might fail to make the proper inference. Experiencing—if not knowing—how thoughts, feelings, behaviors, and circumstances fit together in our own lives, helps us make sense of other people. We term this accumulation of a repertoire of plausible, self-experienced perspectives that facilitates adjustment perspective-pooling.

**Figure 3:**
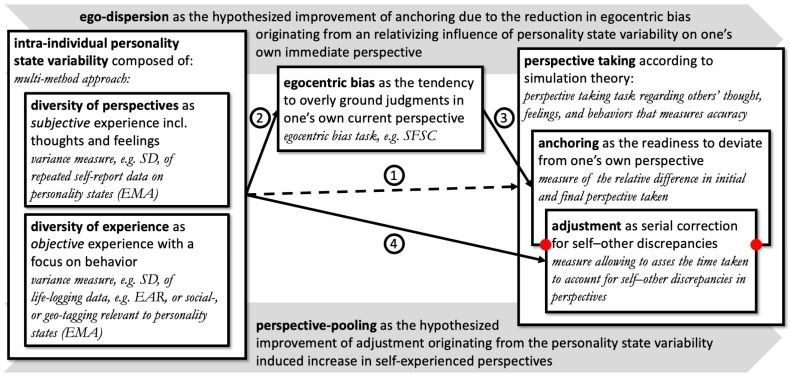
Proposed relation of state variability and perspective taking. *Format*: constructs are in bold, construct specifying notes in roman, and construct operationalizations in italic. *Abbreviations*: EAR—electronically activated recorder; EMA—ecological momentary assessment; SD—standard deviation; SFSC—self-focus sentence completion task. *Numbers*: Compare Section 3.3, summarizing hypotheses (1) to (4). We propose a two-fold positive relation between state variability and perspective taking. Ego-dispersion describes the effect of state variability on anchoring mediated by egocentric bias ((2) and (3)). Perspective-pooling describes a direct effect of state variability on the adjustment process (4). Taken together, this should explain how state variability allows for more accurate perspective taking (1). The solid arrows specify the two routes hypothesized by our model, while the dashed arrow specifies the overall effect if the relation of state variability on perspective taking is considered without the details of our model. Furthermore, the dashed arrow may specify the direct effects of state variability on perspective taking not considered by our model.

Taken together, living through a multitude of personality states positively influences perspective taking, because it implies the experience of a multitude of perspectives, enabling us to distance ourselves from our own perspectives, and to relate to how other people experience the world. Thus, state variability is the starting point of ego-dispersion and perspective-pooling, two functionally independent routes, which jointly benefit the efficiency of simulating someone else’s perspective (cf. Figure 3). In the next two sections we describe these routes in more detail.

### 3.1. Ego-Dispersion Route

With ego-dispersion (cf. Figure 3), we propose that the reoccurring shifts in your own perspective that you experience as your personality states change reduces your egocentric bias. Being aware that your current perspective is transient and only one of many possible perspectives undermines the significance of your own point of view as an anchor when considering another person’s perspective. Therefore, you may be more able or willing to deviate from your own perspective when attributing a perspective to someone else and may make more adjustments before you terminate the simulation.

Consider the spectrum of individual differences in state variability in healthy adults. At one extreme are people with exceptionally high scores in state variability, and on the other are people whose personality states hardly fluctuate. Take the (exaggerated) example of the cliché stoic, an ever-calm philosopher unmoved by what is happening to her. If you were to assess her personality states, she would always report to be cheerful. The stoic perspective centers around the sole state of cheerfulness, for which the price is experiencing only one pleasant perspective despite the ups and downs of life. In contrast, you most likely move regularly through a variety of personality states and experience various perspectives of which cheerfulness is just one which you can easily relate to. Ego-dispersion suggests that state variability counteracts egocentric bias, thus you are less anchored in one particular perspective, because more perspectives are similarly immediate rather than distant to you. To the stoic, only cheerfulness is immediate. To you, there is joy and pain, calm and disquiet (i.e., a wide spectrum of personality states you have experienced more or less recently).

**Figure 4:**
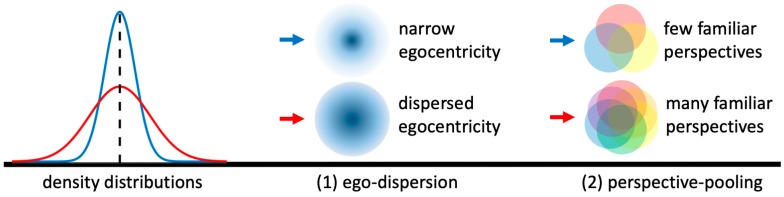
The two links, ego-dispersion and perspective-pooling. With increased state variability (Red > Blue) (1) egocentricity decreases (more blurring of the center of the circle from top to bottom) and (2) the number of self-experienced perspectives availabel for simulation increases (more coloured circles from top to bottom).

There are two ways to put it, people high in state variability are either (a) equally as egocentric as people low in state variability, but they experience more perspectives as “central” (i.e., more immediate to them), or (b) they are less egocentric because state variability interferes with the allocation of what is “central” to them (cf. the blurred center of the circles in (1) in Figure 4). Thus, ego-dispersion is the hypothesized route by which state variability reduces or relativizes the egocentric bias. This weakens anchoring and allows for a greater sensitivity to other cues and information than one’s own perspective when taking someone else’s perspective. Note that ego-dispersion leads to improved accuracy only indirectly, as a weaker egocentric bias implies a reduced anchoring effect on the adjustment process. Because we believe this process to be fairly content-independent, we assume that the effect is domain-general. It matters less how variable you are with respect to which specific aspect of your personality, instead, it is the general awareness of the transiency of your own perspective that is decisive at this stage.

### 3.2. Perspective-Pooling Route

Over time, people high in state variability become familiar with and knowledgeable of a wider range of perspectives. With perspective-pooling (cf. Figure 3), we suggest that this self-knowledge provides valuable inputs that facilitate the efficient simulation of another person’s perspective. Specifically, it allows for relatively faster and more accurate adjustments that reduce existing self–other discrepancies in perspectives. In contrast to ego-dispersion, we believe that this effect is domain-specific (e.g., if you are variable in extraversion but not in conscientiousness, adjustment advantages may apply only insofar as the perspective taking efforts relate to someone else’s social engagement but not to where it concerns their diligence). The difference in domain-specificity may be used as a further means to differentiate perspective-pooling and ego-dispersion.

Consider Figure 4: Red has greater state variability than Blue regarding some trait. Conceptually, Red cannot exploit more fragments of self-knowledge than Blue to simulate someone else’s perspective, because, according to Fleeson’s conceptualization [9], both may have experienced the entire range of state levels. However, a larger range of them seems to be more immediately available to Red (cf. colored circles in (2) in Figure 4). Therefore, Red can—all else being equal—simulate more efficiently than Blue under most circumstances. Eventually, Red is either (a) as equally accurate as Blue but faster, or (b) Red is more accurate than Blue given the same amount of time if Red’s anchor is not preventing the additional accuracy-increasing adjustments. To give another example, imagine you rarely ever experienced feelings of mistrust and you never cared about materialistic things like a marble, simulating how Sally is feeling about her situation with Anne will put you at a disadvantage when compared to someone who has previously suffered materialistic loss and mistrust.

Importantly, perspective-pooling has less to do with egocentric bias than with the richness of experience over time. In other words, perspective-pooling builds on the increased growth in diverse self-knowledge for people high in state variability. Thus, perspective taking should improve with age. However, research indicates that aging has a negative effect on perspective taking. The suggested mechanisms include a loss of motivation and cognitive decline, among others [80,81]. Therefore, as a side note, for people who score high in state variability, we would expect a sharper increase and a flattened decrease of the inverted-U shaped curve for the progression of perspective taking abilities over the life span, because of perspective-pooling. In contrast, we do not expect a similar dynamic with respect to egocentric bias because of ego-dispersion.

### 3.3. Examining the Link between State Variability and Perspective Taking

Assume that all of the relevant variables can be assessed sufficiently. Based on the intuition that the more variable your own daily thoughts, feelings, and behaviors are, the better you might be at inferring the thoughts, feelings, and behaviors of others, our theory suggests the following: (1) Individuals with a higher (vs. lower) state variability should perform better at perspective taking. For testing this, we suggest correlating state variability as the standard deviation of the trait density distributions, and accuracy as self–other discrepancy in perspectives in trait-matched perspective taking tasks. To exclude that the direction of the relation is actually contrary to our prediction, we predict that training individuals in perspective taking should not subsequently increase individuals’ state variability in their everyday live. Note that state variability requires ambulant data collection, but all of the other variables key to the theory can be assessed in a laboratory setting.

With respect to ego-dispersion, (2) individuals with a higher (vs. lower) state variability should be less egocentric, which could be tested by relating state variability to the outcome in (e.g., the self-focus sentence completion task (SFSC) [82]). As in the case of perspective taking, reducing individuals’ egocentric bias though training should not increase their personality state variability. (3) We expect less (vs. more) egocentric individuals to adjust their perspective, not more or less accurate but comparatively more (i.e., we predict a relatively larger difference between initial and final perspectives taken for less egocentric individuals). (4) With respect to perspective-pooling, individuals with a higher (vs. lower) state variability are expected to make more efficient adjustments during perspective taking (i.e., they should account faster for any given self–other discrepancy in perspectives). However, they should only be more accurate in dependence to reduced anchoring. Moreover, (5) we expect ego-dispersion but not perspective-pooling to be effective in a domain-general fashion. Thus, participants’ accuracy but not their relative deviation from their initial perspective may vary in dependence to the trait-specific contents of the task at hand.

Taken together (cf. (1)), if state variability is related to an individual’s perspective taking abilities through both ego-dispersion and perspective-pooling, perspective taking should be more efficient for individuals higher (vs. lower) in state variability. Specifically, they should be comparatively faster and more accurate with respect to tasks whose content relates to traits in which participants are more variable. Alternatively, (6) if perspective-pooling is the only link, perspective taking should be faster but similarly accurate with respect to tasks whose content relates to traits in which participants are more variable. In contrast, if ego-dispersion is the only link, participants higher in state variability should deviate more from their initial perspective but be equally inaccurate independent of trait-related contents of the task. If neither of these predictions applies, support for a positive association of state variability and perspective taking according to our theory is lacking.

## 4. Discussion

In the current paper, we propose a model that builds on state variability as a dynamic aspect of personality that affects our social cognitive abilities. Whereas personality traits describe general affective, behavioral, and cognitive tendencies, state variability captures how often and strongly people diverge from their general dispositions. The more often and strongly they diverge, the more the range of the subjective experiences they have on a more or less regular basis in everyday life expands. With ego-dispersion and perspective-pooling, we argued for two complementary routes, in which the diversity of self-experienced perspectives may increase the likelihood of being able to efficiently infer the perspective of other people. Despite its arguably intuitive appeal, the theory and its empirical investigation face certain limitations and alternative explanations that have to be taken into account, the most important of which are considered in the following sections.

Eventually, in asking whether who we are impacts what we are able to do, we are striving for a more fine-grained integration of personality and ability. We follow a trend in the mind and brain sciences, in order to give a more holistic picture of the human faculty, which considers individuals within the context of their (social) environment. Given how alienating living in a globalized world may be, where people of unfamiliar cultures, ethnicities, and socioeconomic backgrounds are neighbors, it is important to explore how the diversity in our own personalities may propagate mutual understanding through experiences of overlapping or shared perspectives.

### 4.1. Limitations

First, we assume that our predictions apply to any personality trait. However, it is of course possible that state variability is more important in some traits, possibly those relevant to social interaction, than in others or that global cross-trait variability is decisive after all. Moreover, we do not make any claims of whether within- or cross-context state variability [15] is more relevant. Nevertheless, differences are possible. Thus, research is needed to examine whether the relevance of state variability differs across individual traits or contexts.

Second, we argue for a multi-method approach to trait-specific state variability to counterbalance the weaknesses of self-reports and behavioral measures. The current state of the art of operationalizing personality states and state variability, however, may still require more basic research before state variability can be utilized in the proposed manner. Furthermore, using the multi-method approach prevents evaluating the assumption that the diversity of perspectives is indeed relevant over and above the diversity of experiences.

Third, we outline key features of the speed–accuracy perspective taking task, including the assessment of the initially and finally taken perspective, and social inferences with personality trait relevant content including thoughts, feelings, and behaviors. All this may be necessary to differentiate between ego-dispersion and perspective-pooling. Nevertheless, differentiating the two routes empirically with respect to anchoring and adjustment may be difficult, because anchoring and adjustment are closely related and describe foremost a conceptual segmentation of simulation.

Forth, beyond the related psychological factors, like empathy and intelligence, discussed here, there may be other mediators or moderators influencing the relation between state variability and perspective taking that are not part of our theory and that have not been mentioned.

Fifth, the assumed processes may vary across the sub-samples of the population. For example, individuals with specific mental disorders, such as bipolar or borderline personality disorder, may be extremely high in state variability, but in these cases, their experience might not result in beneficial perspective-pooling, because their self-knowledge might not transfer to others. In summary, we tried to balance a comprehensive presentation of the psychological concepts involved and practical recommendations regarding their investigation and the evaluation of our theory.

### 4.2. Alternative Explanations

A potential approach with even greater ecological validity than the method proposed in this paper would further consider the potential variability in perspective, taking performance over time, or following personality state changes.^4^ It is likely that cognitive performance levels are subject to similar fluctuations as personality states levels [83,84,85]. Therefore, it would be interesting to assess the personality state level in which participants undergo perspective taking tasks, and to repeat the task while participants are occupying different state levels. Of course, training effects would have to be considered as well. However, the intraindividual variability of perspective taking abilities is not central to our theory, which focuses on the impact of intraindividual variability of personality states and not the individual, momentary personality state per se.

Future investigations into our theory might, however, consider the influence of self-monitoring on personality states and their variability.^5^ Individuals who score high in self-monitoring voluntarily adapt their self-expression so as to gain appraisal or to protect themselves from the disapproval from others [86,87]. One question might be whether there is a difference between the personality states they “truly” occupy, and the one suggested by their expressive behavior (including self-reports). Moreover, do the continuous social inferences needed for increased self-monitoring lead to improved perspective taking?

Potentially the most interesting alternative explanation may concern the origin of improved perspective taking regarding state variability.^6^ Consider once again Red and Blue’s difference in extraversion; perhaps it is not the overall variability in extraversion that gives Red an advantage over Blue, but simply the relative time spent in more extraverted states than Blue. It may be that Red’s increased social involvement in these situations makes Red more experienced in perspective taking. Thus, it is not the general state variability, including states extremely low and high in extraversion, but only the sub-set of highly extraverted states that affects perspective taking. However, this alternative explanation could only be true when the relevant trait is also assumed to relate directly to perspective taking. Whereas our theory is more trait-agnostic, the alternative explanation is more likely for social traits than for non-social traits and suggests that state variability would only be of relevance where the trait level is as well. Therefore, specific relationships between certain traits and perspective taking abilities add a layer of complexity to the way in which variability across a trait will affect perspective taking. Employing our suggested empirical approach should allow for comparing the outlined alternative explanation and our theory.

## 5. Conclusions

The goal of this paper is to advance ability–personality integration research by means of considering state variability as an additional feature of personality traits. We reviewed recent developments in personality and social cognition research that suggest that we often rely on our own perspectives when inferring other people’s minds. On the one hand, we have suggested that state variability may influence egocentric bias and thus how anchored people are (i.e., the ego-dispersion route). On the other hand, we have proposed that state variability enriches the repertoire of self-experienced perspectives that allow for efficient adjustments (i.e., the perspective-pooling route). Additionally, we discussed other influencing psychological constructs as well as confounds that might distort the proposed relationship. Furthermore, we have suggested hands-on ways to tackle the central hypotheses of our theory experimentally. Thus, we provide the narrative and theoretical background for future investigations to test our theory. Hopefully, we also inspired the reader to think differently about the possible relations and interactions between personality and cognitive abilities, considering not only interindividual but intraindividual differences as well.

## Footnotes

1 The relative variability index can be calculated using free sofware packages for R and MATLAB available online here: https://ppw.kuleuven.be/okp/software/relative_variability/.

2 We are indebted to an anonymous reviewer for making us aware of this article and the issue it raises.

3 We thank the editorial team for their suggestion to raise the issue of behavioral measures and context-effects.

4 We thank the anonymous Reviewer 3 for raising this point.

5 We thank the anonymous Reviewer 2 for raising this point.

6 We thank the anonymous Reviewer 1 for raising this point.

## References

[1] Ackerman P.L. (2018). The Search for Personality–Intelligence Relations: Methodological and Conceptual Issues. J. Intell..

[2] John O.P., Naumann L.P., Soto C.J. (2008). Paradigm shift to the integrative big five trait taxonomy: History, measurement, and conceptual issues. Handbook of Personality: Theory and Research.

[3] Chamorro-Premuzic T., Furnham A. (2003). Personality predicts academic performance: Evidence from two longitudinal university samples. J. Res. Personal..

[4] Ozer D.J., Benet-Martínez V. (2006). Personality and the prediction of consequential outcomes. Annu. Rev. Psychol..

[5] O’Connor M.C., Paunonen S.V. (2007). Big Five personality predictors of post-secondary academic performance. Personal. Individ. Differ..

[6] Roberts B.W., Kuncel N.R., Shiner R., Caspi A., Goldberg L.R. (2007). The Power of Personality: The Comparative Validity of Personality Traits, Socioeconomic Status, and Cognitive Ability for Predicting Important Life Outcomes. Perspect. Psychol. Sci..

[7] Poropat A.E. (2009). A meta-analysis of the five-factor model of personality and academic performance. Psychol. Bull..

[8] Cervone D. (2005). Personality Architecture: Within-Person Structures and Processes. Annu. Rev. Psychol..

[9] Fleeson W. (2001). Toward a Structure- and Process-integrated View of Personality: Traits as Density Distributions of States. J. Personal. Soc. Psychol..

[10] Fleeson W., Jayawickreme E. (2015). Whole Trait Theory. J. Res. Personal..

[11] Fleeson W., Law M. (2015). Trait enactments as density distributions: The role of actors, situations, and observers in explaining stability and variability. J. Personal. Soc. Psychol..

[12] Noftle E.E., Fleeson W. (2010). Age Differences in Big Five Behavior Averages and Variabilities Across the Adult Life Span: Moving Beyond Retrospective, Global Summary Accounts of Personality. Psychol. Aging.

[13] Baird B.M., Le K., Lucas R.E. (2006). On the nature of intraindividual personality variability: Reliability, validity, and associations with well-being. J. Personal. Soc. Psychol..

[14] Jones A.B., Brown N.A., Serfass D.G., Sherman R.A. (2017). Personality and density distributions of behavior, emotions, and situations. J. Res. Personal..

[15] Geukes K., Nestler S., Hutteman R., Küfner A.C.P., Back M.D. (2017). Trait personality and state variability: Predicting individual differences in within- and cross-context fluctuations in affect, self-evaluations, and behavior in everyday life. J. Res. Personal..

[16] Mestdagh M., Pe M., Pestman W., Verdonck S., Kuppens P., Tuerlinckx F. (2018). The relative variability index as a generic mean-corrected variability measure for bounded variables. Psychol. Methods.

[17] Block J. (1961). Ego identity, role variability, and adjustment. J. Consult. Psychol..

[18] Donahue E.M., Robins R.W., Roberts B.W., John O.P. (1993). The divided self: Concurrent and longitudinal effects of psychological adjustment and social roles on self-concept differentiation. J. Personal. Soc. Psychol..

[19] Diehl M., Hastings C.T., Stanton J.M. (2001). Self-concept differentiation across the adult life span. Psychol. Aging.

[20] Suh E.M. (2002). Culture, identity consistency, and subjective well-being. J. Personal. Soc. Psychol..

[21] Campbell J.D., Assanand S., Di Paula A. (2003). The structure of the self-concept and its relation to psychological adjustment. J. Personal..

[22] Baird B.M., Lucas R.E., Donnellan M.B. (2017). The role of response styles in the assessment of intraindividual personality variability. J. Res. Personal..

[23] Deng S., McCarthy D.E., Piper M.E., Baker T.B., Bolt D.M. (2018). Extreme Response Style and the Measurement of Intra-Individual Variability in Affect. Multivar. Behav. Res..

[24] Bock R.D. (1972). Estimating item parameters and latent ability when responses are scored in two or more nominal categories. Psychometrika.

[25] Zettler I., Lang J.W.B., Hülsheger U.R., Hilbig B.E. (2015). Dissociating Indifferent, Directional, and Extreme Responding in Personality Data: Applying the Three-Process Model to Self- and Observer Reports. J. Personal..

[26] Conner T.S., Tennen H., Fleeson W., Barrett L.F. (2009). Experience Sampling Methods: A Modern Idiographic Approach to Personality Research. Soc. Personal. Psychol. Compass.

[27] Shiffman S., Stone A.A., Hufford M.R. (2008). Ecological Momentary Assessment. Annu. Rev. Clin. Psychol..

[28] Csikszentmihalyi M., Larson R. (2014). Validity and Reliability of the Experience-Sampling Method. Flow and the Foundations of Positive Psychology.

[29] Wrzus C., Mehl M.R. (2015). Lab and/or Field? Measuring Personality Processes and Their Social Consequences. Eur. J. Personal..

[30] Mehl M.R., Pennebaker J.W., Crow D.M., Dabbs J., Price J.H. (2001). The Electronically Activated Recorder (EAR): A device for sampling naturalistic daily activities and conversations. Behav. Res. Methods Instrum. Comput..

[31] Mehl M.R., Robbins M.L. (2012). Naturalistic observation sampling: The Electronically Activated Recorder (EAR). Handbook of Research Methods for Studying Daily Life.

[32] Pennebaker J.W., Booth R.J., Boyd R.L., Francis M.E. (2015). Linguistic Inquiry and Word Count: LIWC.

[33] Mehl M.R., Wrzus C., John O.P., Robins R.W. (2018). Ecological sampling methods for studying personality in daily life. The Handbook of Personality.

[34] Rauthmann J.F., Gallardo-Pujol D., Guillaume E.M., Todd E., Nav C.S., Sherman R.A., Ziegler M., Jones A.B., Funder D.C. (2014). The situational Eight DIAMONDS: A taxonomy of major dimensions of situation characteristics. J. Personal. Soc. Psychol..

[35] Rauthmann J.F., Sherman R.A. (2015). Ultra-Brief Measures for the Situational Eight DIAMONDS Domains. Eur. J. Psychol. Assess..

[36] Davis M.H. (2018). Empathy: A Social Psychological Approach.

[37] Preckel K., Kanske P., Singer T. (2018). On the interaction of social affect and cognition: Empathy, compassion and theory of mind. Curr. Opin. Behav. Sci..

[38] Kanske P., Böckler A., Trautwein F.-M., Singer T. (2015). Dissecting the social brain: Introducing the EmpaToM to reveal distinct neural networks and brain-behavior relations for empathy and Theory of Mind. NeuroImage.

[39] Tusche A., Böckler A., Kanske P., Trautwein F.-M., Singer T. (2016). Decoding the Charitable Brain: Empathy, Perspective Taking, and Attention Shifts Differentially Predict Altruistic Giving. J. Neurosci. Off. J. Soc. Neurosci..

[40] Winter K., Spengler S., Bermpohl F., Singer T., Kanske P. (2017). Social cognition in aggressive offenders: Impaired empathy, but intact theory of mind. Sci. Rep..

[41] Westra E. (2017). Character and theory of mind: An integrative approach. Philos. Stud..

[42] Blair R.J.R. (2005). Responding to the emotions of others: Dissociating forms of empathy through the study of typical and psychiatric populations. Conscious. Cogn..

[43] Sonnby-Borgström M., Jönsson P., Svensson O. (2003). Emotional Empathy as Related to Mimicry Reactions at Different Levels of Information Processing. J. Nonverbal Behav..

[44] Sonnby–Borgström M. (2002). Automatic mimicry reactions as related to differences in emotional empathy. Scand. J. Psychol..

[45] Chartrand T.L., Bargh J.A. (1999). The chameleon effect: The perception-behavior link and social interaction. J. Personal. Soc. Psychol..

[46] Krauss R.M., Glucksberg S. (1977). Social and Nonsocial Speech. Sci. Am..

[47] Epley N., Keysar B., Van Boven L., Gilovich T. (2004). Perspective taking as egocentric anchoring and adjustment. J. Personal. Soc. Psychol..

[48] Apperly I.A., Warren F., Andrews B.J., Grant J., Todd S. (2011). Developmental Continuity in Theory of Mind: Speed and Accuracy of Belief–Desire Reasoning in Children and Adults. Child Dev..

[49] Coburn P.I., Bernstein D.M., Begeer S. (2015). A new paper and pencil task reveals adult false belief reasoning bias. Psychol. Res..

[50] Todd A.R., Forstmann M., Burgmer P., Brooks A.W., Galinsky A.D. (2015). Anxious and egocentric: How specific emotions influence perspective taking. J. Exp. Psychol. Gen..

[51] Dziobek I., Fleck S., Kalbe E., Rogers K., Hassenstab J., Brand M., Kessler J., Woike J.K., Wolf O.T., Convit A. (2006). Introducing MASC: A Movie for the Assessment of Social Cognition. J. Autism Dev. Disord..

[52] Baron-Cohen S., Leslie A.M., Frith U. (1985). Does the autistic child have a “theory of mind”?. Cognition.

[53] Parsons S., Mitchell P. (2002). The potential of virtual reality in social skills training for people with autistic spectrum disorders. J. Intellect. Disabil. Res..

[54] Kandalaft M.R., Didehbani N., Krawczyk D.C., Allen T.T., Chapman S.B. (2013). Virtual Reality Social Cognition Training for Young Adults with High-Functioning Autism. J. Autism Dev. Disord..

[55] Tamir D.I., Mitchell J.P. (2013). Anchoring and adjustment during social inferences. J. Exp. Psychol. Gen..

[56] Shanton K., Goldman A. (2010). Simulation theory. Wiley Interdiscip. Rev. Cogn. Sci..

[57] Goldman A.I. (2006). Simulating Minds: The Philosophy, Psychology, and Neuroscience of Mindreading.

[58] Greenwald A.G. (1980). The totalitarian ego: Fabrication and revision of personal history. Am. Psychol..

[59] Eyal T., Steffel M., Epley N. (2018). Perspective mistaking: Accurately understanding the mind of another requires getting perspective, not taking perspective. J. Personal. Soc. Psychol..

[60] O’Connell G., Christakou A., Chakrabarti B. (2015). The role of simulation in intertemporal choices. Front. Neurosci..

[61] O’Connell G., Hsu C.-T., Christakou A., Chakrabarti B. (2018). Thinking about others and the future: Neural correlates of perspective taking relate to preferences for delayed rewards. Cogn. Affect. Behav. Neurosci..

[62] Soutschek A., Ruff C.C., Strombach T., Kalenscher T., Tobler P.N. (2016). Brain stimulation reveals crucial role of overcoming self-centeredness in self-control. Sci. Adv..

[63] Dimaggio G., Lysaker P.H., Carcione A., Nicolo G., Semerari A. (2008). Know yourself and you shall know the other… to a certain extent: Multiple paths of influence of self-reflection on mindreading. Conscious. Cogn..

[64] Shaw D.J., Czekóová K., Pennington C.R., Qureshi A.W., Špiláková B., Salazar M., Brázdil M., Urbánek T. (2018). You ≠ me: Individual differences in the structure of social cognition. Psychol. Res..

[65] Hojat M., Zuckerman M., Magee M., Mangione S., Nasca T., Vergare M., Gonnella J.S. (2005). Empathy in medical students as related to specialty interest, personality, and perceptions of mother and father. Personal. Individ. Differ..

[66] Magalhães E., Costa P., Costa M.J. (2012). Empathy of medical students and personality: Evidence from the Five-Factor Model. Med. Teach..

[67] Costa P., Alves R., Neto I., Marvão P., Portela M., Costa M.J. (2014). Associations between medical student empathy and personality: A multi-institutional study. PLoS ONE.

[68] Melchers M.C., Li M., Haas B.W., Reuter M., Bischoff L., Montag C. (2016). Similar Personality Patterns Are Associated with Empathy in Four Different Countries. Front. Psychol..

[69] Song Y., Shi M. (2017). Associations between empathy and big five personality traits among Chinese undergraduate medical students. PLoS ONE.

[70] Davis M. (1980). A multidimensional approach to individual differences in empathy. JSAS Cat. Sel. Doc. Psychol..

[71] Ziegler M., Danay E., Heene M., Asendorpf J., Bühner M. (2012). Openness, fluid intelligence, and crystallized intelligence: Toward an integrative model. J. Res. Personal..

[72] McGrew K.S. (2009). CHC theory and the human cognitive abilities project: Standing on the shoulders of the giants of psychometric intelligence research. Intelligence.

[73] Raine A., Reynolds C., Venables P.H., Mednick S.A. (2002). Stimulation seeking and intelligence: A prospective longitudinal study. J. Personal. Soc. Psychol..

[74] DeYoung C.G., Peterson J.B., Higgins D.M. (2005). Sources of Openness/Intellect: Cognitive and Neuropsychological Correlates of the Fifth Factor of Personality. J. Personal..

[75] Cattell R.B. (1987). Intelligence: Its Structure, Growth and Action.

[76] Prentice M., Jayawickreme E., Fleeson W. (2018). Integrating whole trait theory and self-determination theory. J. Personal..

[77] Arntz A., Bernstein D., Oorschot M., Schobre P. (2009). Theory of mind in borderline and cluster-C personality disorder. J. Nerv. Ment. Dis..

[78] Ibanez A., Huepe D., Gempp R., Gutiérrez V., Rivera-Rei A., Toledo M.I. (2013). Empathy, sex and fluid intelligence as predictors of theory of mind. Personal. Individ. Differ..

[79] Liepmann D., Beauducel A., Brocke B., Amthauer R. (2007). Intelligenz-Struktur-Test 2000 R, 2 Erweiterte und Überarbeitete Auflagw [Intelligence-Structure-Test 2000 R, 2nd Extended and Revised Edn].

[80] Maylor E.A., Moulson J.M., Munce A.-M., Taylor L.A. (2010). Does performance on theory of mind tasks decline in old age?. Br. J. Psychol..

[81] Duval C., Piolino P., Bejanin A., Eustache F., Desgranges B. (2011). Age effects on different components of theory of mind. Conscious. Cogn..

[82] Exner J.E. (1973). The Self Focus Sentence Completion: A study of egocentricity. J. Personal. Assess..

[83] Avenanti A., Minio-Paluello I., Bufalari I., Aglioti S.M. (2009). The pain of a model in the personality of an onlooker: Influence of state-reactivity and personality traits on embodied empathy for pain. NeuroImage.

[84] Knowles M.L. (2014). Social rejection increases perspective taking. J. Exp. Soc. Psychol..

[85] Bukowski H., Samson D. (2016). Can emotions influence level-1 visual perspective taking?. Cogn. Neurosci..

[86] Snyder M. (1974). Self-monitoring of expressive behavior. J. Personal. Soc. Psychol..

[87] Gangestad S.W., Snyder M. (2000). Self-monitoring: Appraisal and reappraisal. Psychol. Bull..

